# Relationship between Type-D Personality, Physical Activity Behaviour and Climacteric Symptoms

**DOI:** 10.1186/s12905-015-0176-3

**Published:** 2015-02-22

**Authors:** Erika Borkoles, Nick Reynolds, Chantal F Ski, Lilly Stojanovska, David R Thompson, Remco CJ Polman

**Affiliations:** Institute of Sport, Exercise and Active Living, Victoria University, Melbourne, Australia; Centre for the Heart and Mind, Australian Catholic University, Melbourne, Australia; Centre for Chronic Disease Prevention and Management, Victoria University, Melbourne, Australia; Psychology Department, Bournemouth University, Bournemouth, UK

## Abstract

**Background:**

A number of factors have been identified which might influence the variation observed in climacteric symptoms in peri- and post-menopausal women. We examined the role of the distressed or Type-D personality and mode of physical activity or exercise on the climacteric symptoms experienced by peri- or post-menopausal women.

**Methods:**

213 Women (M age 52.2 years, SD = 5.9), 58% classified as peri- and 42% as post-menopausal completed a questionnaire pack consisting of demographic questions, the DS14 (Type-D personality), Kaiser Physical Activity Survey (assessing household care giving, occupational, active living and sport and exercise index) and the Greene Climacteric Scale (Psychological, somatic/physical, vasomotor and sexual symptoms).

**Results:**

Type-D personality and increased levels of household care-giving physical activity were both associated with increased bothersomness for all four climacteric factors. Increased levels of sport and exercise participation on the other hand resulted in less psychological, somatic/physical and sexual functioning problems whereas the active living index was inversely related to somatic/physical climacteric symptoms. Finally, lower income was associated with more psychological and somatic/physical symptoms and being peri-menopausal resulted in more vasomotor symptoms.

**Conclusions:**

The results suggest that mode of physical activity is an important moderator in alleviating climacteric symptoms. In addition, our results support previous findings in that Type-D personality is associated with negative health outcomes. In particular menopausal women with Type-D personality would benefit from interventions (coping, mindfulness training) and regular sport and exercise participation to reduce climacteric symptomology.

**Electronic supplementary material:**

The online version of this article (doi:10.1186/s12905-015-0176-3) contains supplementary material, which is available to authorized users.

## Background

There is considerable evidence now that about 75% of all women experience to varying degree a number of physical and psychological symptoms during the menopausal transition [1,2]. This has been referred to as the climacteric or menopausal syndrome and include psychological (e.g., nervousness, irritability), vasomotor (e.g., hot flashes, night sweats), somatic/physical (e.g., insomnia, headaches, paresthesia) and sexual symptoms (e.g., vaginal dryness, loss of interest in sex) [3]. Significant variation in the experience of menopausal symptoms has been reported in the literature with some women reporting severe, whilst others report minimal physical and psychological symptoms [4]. In a Finish study only 5% of women between the ages of 52 to 56 years were completely asymptomatic; in comparison to 11% of women in the same age range who report severe symptoms [5]. It is still unclear who is going to be affected and why, and more importantly at what stage of the menopausal process the symptoms are most bothersome. In this respect the peri-menopausal stage, the transition from reproductive to non-reproductive has been associated with more severe symptomology compared to post-menopause, when menstruation has ceased permanently [6].

Factors which might explain the variation in the experience of the climacteric syndrome include appraisal, lifestyle (e.g., exercise and sport participation), the presence of mental health problems (e.g., depression), context (e.g., social interactions, socio-cultural) and personality. Few studies have examined the role of personality on symptom severity. Elavsky and McAuley [7] found that trait anxiety and optimism influenced psychological and vasomotor symptoms (VMS) reporting and improvements in physical fitness was also associated with reduced symptomatology.

More recently the distressed or Type-D personality [8] has been indicated as influencing health in normal and chronic disease populations [9,10]. Type-D personality is characterized by negative affectivity (NA) and an inability to express these emotions or behaviors in social interactions (SI; social inhibition) [8]. Type-D Individuals scoring high on NA and SI have been found to experience more chronic stress, social and emotional problems and adverse health events [11,12]. Type-D personality appears to be a personality type which could influence the experience of menopausal symptoms but to date no study has examined this proposition.

There has been significant interest in the role of physical activity (PA) and exercise on weight and bone health in menopausal women and as an alternative option to menopause hormone therapy (MHT) [11]. The evidence for the role of PA and exercise on the experience of climacteric symptoms has been equivocal. In addition, relatively few studies have examined the role of different modes of PA and exercise on climacteric symptomology. When women get older they are less likely to participate in organized exercise and sport but more likely to engage in housework, caregiving and occupational activities [12,13]. As such there is likely to be an underestimation of PA patterns of women. In addition, climacteric complaints might be influenced differently depending on the nature of the activity. However, this has not been examined to date.

The aim of this study was to examine the role of Type-D personality and different modes of PA and exercise behavior (household, occupational, active living, sport and exercise) on the climacteric symptoms experienced by peri- and post-menopausal women. In particular, we examined whether Type-D personality was associated with more or less severe climacteric symptoms and whether the level of participation in active living or sport and exercise influenced climacteric symptoms for peri- and post-menopausal women.

## Methods

### Participants

Women were recruited via advertisements in print and online media and forums (e.g., Australian Menopause Society) through leaflets and researchers attending specific events. Inclusion criteria consisted of women either being in the peri-menopausal (defined as the period from the commencement of menstrual irregularity to one year after the cessation of menstrual periods) or post-menopausal (defined as one year or more after the cessation of menstrual periods) stage [14] and English speaking. Exclusion criteria for the study were no current MHT or in the last 6 months, or any serious medical condition (e.g. cancer or depression). Participants provided written (hardcopy) consent or ticked the appropriate box on the online version and the study was approved by Victoria University Human ethics committee.

### Materials and procedure

Participants completed a questionnaire pack (see Additional file 1), either online or in paper format with return envelope, consisting of a number of demographic questions including age, income, highest educational qualification, medical procedures influencing menopause, presence of chronic or debilitating illness, and the use of psychoactive substances and their personality, smoking, physical activity habits and menopausal symptoms. Completion of the questionnaire pack took approximately 30 minutes.

The DS14 [8] provides a taxonomic and continuous assessments of distressed personality by measuring the traits of negative affectivity (NA, 7-items; e.g., “I often feel unhappy”) and social inhibition (SI, 7-items; e.g., “I am a closed kind of person”). Participants respond using a 5-point Likert scale anchored at 0 = false to 4 = true. A score of 10 or more on both the NA and SI scale indicates the likelihood the respondent fits the Type-D personality profile [15]. The DS14 total score (α = .93) and subscale scores (NA, α = .90; SI, α = .91) were internally consistent in the present sample, in accordance with previous studies [8].

The 21-item Greene Climacteric Scale (GCS) [3] measures the bothersomeness of symptoms associated with menopause, including psychological (depression, anxiety), somatic/physical (e.g., headaches, joint pain), vasomotor (e.g., hot flushes, sweating at night) sexual (lost interest in sex) symptoms. Respondents indicate how bothersome they have found each symptom using a five point Likert scale which ranges from 0 = “Not at all” bothersome to 3 = “extremely” bothersome. Item ratings are summated to provide an overall index of symptom bothersomeness, and summated by subscale to produce symptom domain scores, with high scores indicating bothersome symptoms. The GCS has a replicable factorial structure, adequate test retest reliability [3], and has been normed in Australian samples [16]. The GCS total scale and subscales were adequately reliable in the present sample (somatic α = .83; vasomotor α = .89; psychological, α = .76; GCS total, α = .90).

The Kaiser Physical Activity Survey is a 75 item survey, adapted from the Baecke Physical Activity Questionnaire [17] designed to measure the habitual activity of females in the domains of paid employment (occupational index), unpaid domestic work and care giving (household care-giving index), daily routine (active living index), and recreational activity (sport and exercise index) [13]. KPAS subscale scores are adjusted for exercise intensity [18] with increasing scores signifying rising levels of physical activity. The KPAS total and subscale scores correlate highly with alternative self-report measures and moderately with objective measures of physical activity and appear to provide a reliable indicator of habitual activity, with one month test retest coefficient being high (0.91) [18].

#### Analysis strategy and statistics

We first examined the data for multicolinearity, normality and outliers. Tolerance was > .10 and the variance inflation factor < 10 for each analysis indicating no problems related to multicolinearity. The probability plot of the regression standardised residuals and the scatter plots suggested no violation of normality and an absence of outliers. The latter was confirmed by inspecting the Mahalanobis distances.

Differences between the peri- and post-menopausal women on the GCS and KPAS were explored using multivariate analysis of variance (MANOVA). In addition, we explored whether Type-D individuals reported different PA and exercise levels using MANOVA. In the instance of a significant main effect we conducted analysis of variance (ANOVA) to explore differences.

Regression models were used to analyse the influence of PA behaviour and Type-D personality on menopausal bothersomeness of symptoms (psychological, somatic, vasomotor, sexual functioning). We controlled for menopausal status (peri- or postmenopausal), income, educational achievement, and smoking at step one. At step two the four subscales of the KPAS (occupational, household care-giving, active living, sport and exercise) and Type-D (participants classified as Type-D or not Type-D) were entered. In the instance of a significant effect for any of the KPAS factors we conducted further regression analysis by examining whether there was an interaction effect for KPAS factors with menopausal status. In this additional step we entered the multiplication of the dummy coded menopausal status variable with the KPAS factor which was a significant predictor. Significance was set at P < .05 and SPSS version 22 was used for data analysis (IBM, Somers, NY).

## Results

213 Women ($\overline{\mathrm{x}}$ age = 52.2 years, SD = 5.9; age range 36–67 years) completed the questionnaire pack of which 125 (58%; $\overline{\mathrm{x}}$ age = 50 years, SD = 4.2) were classified as peri-menopausal and 88 (42%; $\overline{\mathrm{x}}$ age = 54.2 years, SD = 5.9) as post-menopausal. The sample included 17 participants who had undergone a surgical procedure that might affect their experience of menopause. Two postmenopausal women had each undergone a hysterectomy with bilateral oophorectomy, and 15 peri-menopausal participants had undergone a hysterectomy (n = 8) or hysterectomy with unilateral oophorectomy (n = 7). The sample contained no reports of chronic and debilitating illness and no psychoactive substance use was reported.

Most participants were Caucasian (89%) and employed on either a full- (70.5%) or part-time (21.5%) bases, possessed a tertiary qualification (Secondary school = 14.6%; Trade diploma = 20.3%; University degree = 30.1%; Post-graduate degree = 35.6%), relatively affluent (< $20.000 = 5.45%; $21-40.000 = 12.3%; $41-60.000 = 25%; $61-80.000 = 20.8%; > $81.000 = 36.8%), and had one or more children (0 = 23.3%; 1 = 12.6%; 2 = 42.2%; 3 ≥ 22%). There were 64 (30.3%) women classified as Type-D and 147 (69.7%) as non Type-D.

**Figure 1:**
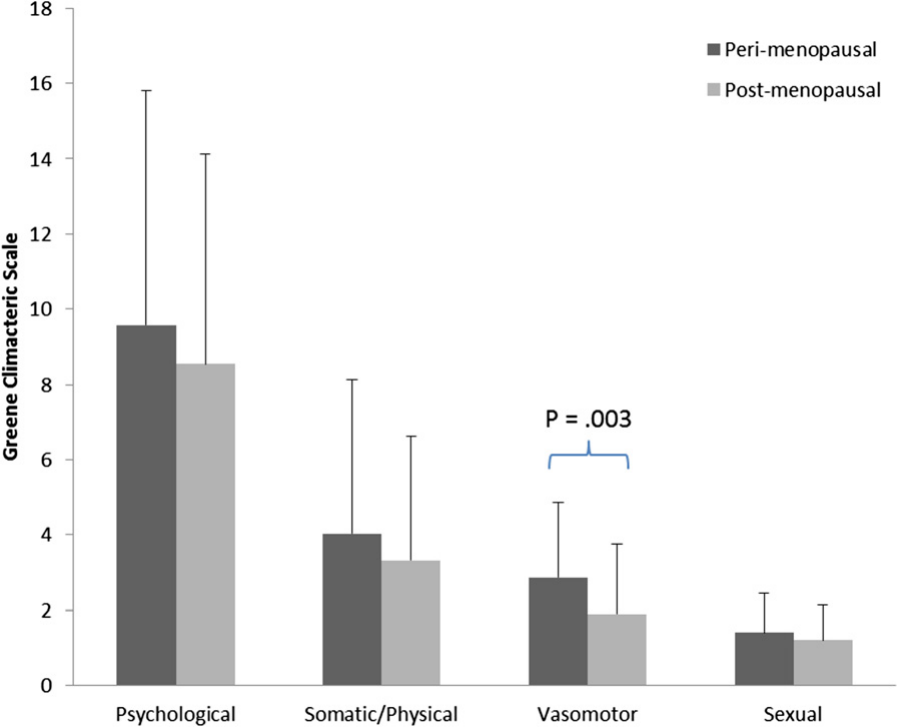
**Mean and standard deviation for the GCS sub-scales for the peri- and post-menopausal women.**

**Figure 2:**
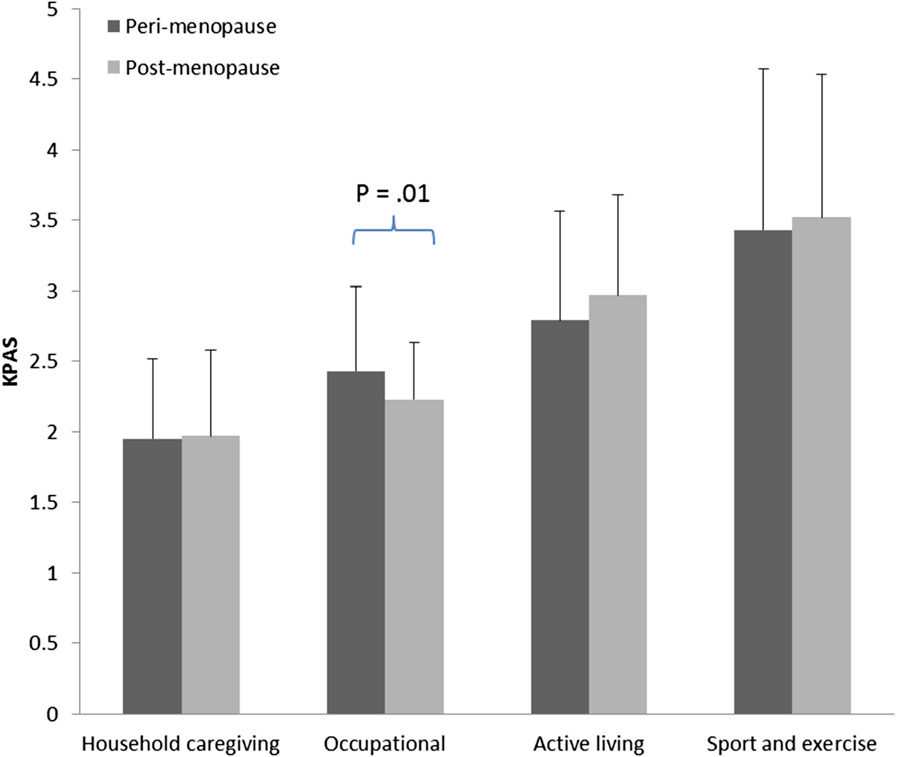
**Mean and standard deviations of the KPAS physical activity subscales for the peri- and post-menopausal women.**

The MANOVA for menopausal status was significant (Wilks’ λ = .91; P = .02; Eta^2^ = .094). Follow-up ANOVA showed a significant difference for VMS (F(1,191) = 9.33; P = .003; Eta^2^ = .046) and occupational activity index of the KPAS (F (1,194) = 7.07; P = .01; Eta^2^ = .039) with the peri-menopausal women reporting greater VMS and more occupational activity (see Figures 1 and 2).

The MANOVA for Type-D personality and KPAS factors was also significant (Wilks’ λ = .94; P = .03; Eta^2^ = .06). Follow-up ANOVA showed significant effects for household care-giving (F (1,184) = 5.36; P = .02; Eta^2^ = .03) and occupation (F (1,184) = 5.31; P = .03; Eta^2^ = .03) but not active living (P = .08) or sport and exercise (P = .11). Type-D women spend more time in household care giving (2.07 vs. 1.85) and occupational (2.51 vs. 2.31) activity compared to non Type-D women.

**Figure 3:**
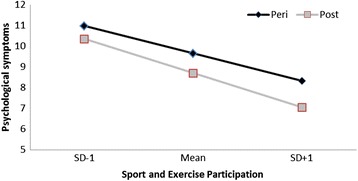
**Interaction effect for menopausal status and sport and exercise participation in relation to psychological symptoms.**

Table 1 provide an overview of the results of the regression analysis for the four factors of the GCS. For all four factors of the GCS (psychological, somatic, vasomotor, sexual functioning) higher levels of household care-giving and being classified as Type-D was associated with increased bothersomeness of symptoms and decreased sexual functioning. In addition, increased levels of active living were associated with increased somatic symptoms. Increased sport and exercise participation on the other hand resulted in decreased somatic symptoms and increased sexual functioning. For the psychological symptoms there was also an interaction with menopausal status. The post-menopausal women showed lower levels of psychological symptoms at higher levels of sport and exercise participation in comparison to the peri-menopausal women (see Figure 3).

**Table 1:** **Result of the regression analysis**

**Step and variable**	**Beta**	**R** ^**2**^	**ΔR** ^**2**^
Dependent variable: **Psychological**			
Step 1: Income	-.224	.090^**^	
Step 2: Household care-giving	.178^**^		.287^***^
Type-D personality	.450^***^		
Step 3: Sport and Exercise x Menopausal status	-.470^*^		.021^*^
Dependent variable: **Somatic/Physical**			
Step 1: Income	-.345^***^	.118^***^	
Step 2: Household care-giving	.258^***^		.226^***^
Active living	.204^**^		
Sport and exercise	-.224^**^		
Type-D personality	.321^***^		
Dependent variable: **Vasomotor**			
Step 1: Menopausal status	-.207^**^	.102^***^	
Step 2: Household care-giving	.176^*^		.075^*^
Type-D personality	.161^*^		
Dependent variable: **Sexual functioning**			
Step 1: N.S.		.044	
Step 2: Household care-giving	.189^**^		.134^***^
Sport and Exercise	-.206^**^		
Type-D personality	.216^**^		

Only significant predictors are included. Note: * = p < .05; ** = p < .01; *** = p < .001.

## Discussion

This study examined the role of Type-D personality and different modes of PA and exercise behaviour on the climacteric symptoms experienced by peri- and post-menopausal women. Both Type-D personality and increased levels of household care-given resulted in increased bothersomness for all four climacteric factors. Supporting previous findings, increased levels of sport and exercise participation reduced psychological and somatic/physical symptoms as well as sexual symptoms in all women whereas active living was inversely related to somatic/physical climacteric symptoms. Lower income was associated with more psychological and somatic/physical symptoms and menopausal status only influenced VMS.

To our knowledge this is the first study which has examined the role of different modes of PA and exercise behaviour of peri- and post-menopausal women on climacteric symptomology. Our results are in line with previous findings suggesting that regular sport and exercise participation alleviate somatic/physical symptoms [1,19,20]. Evidence from randomized control trials [21,22] and systematic reviews [11,19,23] on the efficacy of exercise for menopausal symptoms provides support for regular PA and exercise in alleviating the psychological and somatic/physical symptoms of the climacteric syndrome. For example Haimov-Kochman et al. [24] found a dose–response relationship between climacteric complaints and exercise behavior with higher exercise frequency resulting in reduced psychological and somatic/physical complaints but not VM or sexual symptoms. Ivarsson et al. [25] on the other hand found that women who were more physically activity had lower severity and shorter duration of VMS compared to more sedentary women. Others also reported that physically active women reported fewer menopausal symptoms than active women [20]. On the contrary, Whitcomb et al. [26] found that increased physical activity was associated with increased VMS (hot flashes).

Our results indicate that sport and exercise participation can also result in reduced sexual symptoms but does not influence VMS this despite exercise interventions usually target women who report high frequency of VMS, and measure its effectiveness based on change in symptom frequency. However, PA related to household care-giving resulted in a significant increase in bothersomeness in VMS and all other climacteric domains. The household and care-giving section of the KPAS examines activities related to looking after children, preparing meals, cleaning, shopping, yard work, heavy outdoor work and major home decoration or repair activities. It appears that the physical activity involved in such activities do not counteract the stressors involved in dealing with these day to day hassles.

Surprisingly increased levels of active living were related to more somatic/physical complaints. The active living scale of the KPAS assesses activities like active transport to work or walking and biking as a leisure activity. A possible explanation for the inverse relationship between somatic/physical symptoms and active living is that the intensity of the active living activities was not sufficient enough to provide health benefits. However, studies using objective measurement of PA and exercise behaviour would be required to examine this further.

The role of sport and exercise participation on climacteric symptoms has been equivocal. Our result support previous research and demonstrates that higher levels of sport and exercise participation alleviate general menopausal symptoms, including psychological and somatic/physical [23]. Moreover, the psychological benefits appear to be greater in postmenopausal women with higher levels of sport and exercise participation than in peri-menopausal women. In addition, our data suggest reduction in sexual symptoms with increased levels of sport and exercise participation but we found no relationship with VMS.

A number of explanations have been provided how exercise and sport participation can directly and indirectly improve psychological and somatic/physical well-being during menopause. These include biochemical (endorphins, serotonin, noradrenaline), physiological (increase in core body temperature) and psychosocial (self-esteem, self-efficacy, personal control, time-out) explanations [27]. However, it is likely that the benefits of exercise and sport are due to an interaction of different physiological, biochemical and psychological factors and its impact fluctuate depending on age, exercise history, mode, frequency and intensity. This is supported by our findings that PA in different domains has varied influence on climacteric symptomology and possibly explains why postmenopausal women benefit more from sport and exercise participation in reducing psychological symptoms than peri-menopausal women. From a practical perspective regular sport and exercise participation should be encouraged in menopausal women [28] to reduce symptomology whereas household caregiving activities should be limited where possible.

To date few studies have examined the role of personality on menopausal symptomology. Our study indicates that Type-D personality is associated with higher levels of bother across all domains of the climacteric syndrome. Grounded in psychological theory [29] Type-D personality is distinct from the five-factor framework of personality [30]. Type-D personality was initially developed in cardiovascular patients in whom it was shown to be predictive of negative health outcomes [31,32]. More recent studies have also indicated that Type-D personality is associated with negative health outcomes in the general population including more mental health problems and poorer physical health status [10]. In addition, Type-D individuals have been shown to respond with greater cardiovascular and neuroendocrine reactivity to laboratory stressors, [33] as well as experiencing more psychosocial stressors [34]. The current findings would suggest that Type-D personality is also associated with increased climacteric symptomology in peri- and postmenopausal women.

Type-D personality has also been associated with increased health care utilisation [34] and negative health behaviours like having a poor diet, smoking and less physical activity [35]. The current study did not find significant differences in active living or sport and exercise behaviours between those classified as Type-D or non Type-D. In addition, no difference was apparent in smoking behaviour.

Although Type-D has been shown to be heritable [36] and stable construct [37] there are opportunities for non-pharmacological interventions to reduce the negative symptoms associated with Type-D personality. For example, research has indicated that Type-D personality is associated with maladaptive coping [38]. As such coping interventions may help women with Type-D personality to better deal with climacteric symptoms. Such interventions could in particular target the appraisal process through cognitive restructuring, development of emotion-focused coping skills to down regulate their emotional state (e.g., breathing) whilst reducing maladaptive avoidance coping strategies. In addition, a mindfulness-based stress reduction training program has been shown to reduce NA and SI in healthy individuals [39].

We used smoking habits as a co-variate because smoking has been associated with increased vasomotor symptoms [40] and reduced PA and exercise participation [41]. Similarly, income and education also have been shown to influence menopausal symptoms [42] and PA and exercise behaviour with higher incomes and increased educational achievements associated with reduced symptoms and higher levels of PA and exercise participation [41]. Only income was related with climacteric factors as assessed by the GCS in the present study. Higher income was associated with decreased psychological and somatic symptoms.

Both income and educational achievement have been associated with increased PA participation [41,43]. In 2007–08 in Australia, adults living in the lowest income households were less likely than those in the higher income households to meet the recommended physical activity guidelines (28% compared with 42%). Furthermore, only 26% of women living in the lowest income households exercised compared to 53% in the highest income households [44]. Higher levels of PA participation, in turn, result in better physical and mental health [27]. As such our results are not unexpected and suggest that higher income is more important than educational achievement in reducing menopausal general symptomology. Although a recent Norwegian study suggested that higher levels of educational attainment also results in decreased frequency of VMS [42].

Smoking has been found to influence menopausal symptoms in some studies [42,45,46] whereas other studies have found no relationship [47,48]. Possible reasons why smoking was not related with climacteric symptoms in the present study sample was that they were relatively affluent and highly educated and consisted of very few smokers (11.2%).

Menopausal status only influenced VMS. Peri-menopausal women experienced more severe VMS than the postmenopausal women. The literature on VMS often equates increased frequency of symptoms with increased bother. Although post-menopausal women might experience VMS more frequently (i.e., hot flashes) [42,49,50] factors like age, general health, sleep symptom sensitivity and affect are more important predictors over and above frequency [48]. In particular, younger age was associated with increased bothersomeness. As such peri-menopausal women might experience fewer symptoms but they might be higher in bothersomeness.

Our study is not without limitations. In particular, our cross-sectional design cannot support causal inferences. Also, data are self-reported which can result in bias (e.g. over-reporting of sport and exercise activities). Finally, our sample consisted of mainly Caucasian women who were relatively affluent and well educated.

## Conclusions

Our study provides evidence that demographic (income), lifestyle (sport and exercise) and personality (Type-D) factors influence the experience of climacteric symptoms in peri- and post-menopausal women. In particular, this is one of the first studies showing that different modes of PA and exercise behaviour influences climacteric symptoms differently. Higher levels of sport and exercise behaviour were associated with reduce psychological, somatic/physical and sexual symptoms whereas household caregiving was associated with increased symptoms across all four climacteric factors. In addition, active living was inversely related to somatic symptoms. Also, those women who are classified as Type-D experience higher levels of climacteric symptoms. Therefore, menopausal women with Type-D personality would benefit from interventions aiming to reduce climacteric symptoms. Such interventions could include coping and mindfulness training as well as the recommendation to engage in regular sport and exercise. However, randomized control trials are needed to test the efficacy of such interventions.

## Additional file

Additional file 1:
**MenopauseQpack.**

## Abbreviations

ANOVA: Analysis of variance
GCS: Greene climacteric scale
KPAS: Kaiser physical activity survey
MHT: Menopause hormone therapy
MANOVA: Multivariate analysis of variance
NA: Negative affectivity
PA: Physical activity
SI: Social inhibition
VM: Vasomotor
VMS: Vasomotor symptoms
