# Supplementary material for: Relationship between Type-D Personality, Physical Activity Behaviour and Climacteric Symptoms
Source: BMC Womens Health. 2015 Feb 22;15:18. doi: 10.1186/s12905-015-0176-3 (PMC4344794; doi:10.1186/s12905-015-0176-3)
Supplement: Additional file 1: — MenopauseQpack. [file 12905_2015_176_MOESM1_ESM.pdf]

Menopause and Exercise Questionnaire Pack

Dear Participant,

Thank you for agreeing to participate in this study on “Exercise Behaviours during Menopause”

Your contribution is much appreciated and highly valued in helping to understand people’s feelings and coping strategies when trying to exercise during and post menopause.

This pack contains a series of short questionnaires that should take you approximately 30-40 minutes to complete. The range of questionnaires is designed to obtain as wide a view as possible towards understanding an individual’s own feelings towards the process of trying to be physically active during menopause.

The questionnaires work best if you are able to answer every question. However, if there is anything you would rather not answer, we respect that. Before you respond, please read the information sheet to participants and the consent form (attached in the link). If you meet the criteria for this research, which is stated in the attached document and willing to participate and share your views with us, please proceed in answering the questions.

We assure you that the information you provide will be kept confidential and anonymous and we will always follow the law to protect you and the information you provide.

Eligibility Questions

Thank you for agreeing to participate in this survey. We have a few preliminary questions relating to your menopausal status.

1. Would you be classified as peri-menopausal? This would mean that your periods have become irregular or have stopped completely within the last 12 months?

- Yes
- No

2. Would you be classified as post-menopausal? This means that it has been at least 12 months since your last period.

- Yes
- No

3. Are you currently receiving Hormone Replacement Therapy?

- Yes
- No

4. Have you received Hormone Replacement Therapy in the last 6 months?

- Yes
- No

5. Do you have a serious medical condition (e.g. cancer)?

- Yes
- No

6. Do you have a chronic psychological condition (e.g. Depression)?

- Yes
- No

Demographical Information

What is your age in years?

____

7. Are you of Aboriginal and/or Torres Strait islander descent?

- Yes
- No

8. Were you born in Australia?

- Yes
- No

9. Are you an Australian citizen?

- Yes
- No

10. In which State do you live?

- ACT
- QLD
- VIC
- NSW
- SA
- WA
- NT
- TAS

11. How would you describe your cultural ancestry? (Choose all that apply)

- Anglo-Celtic
- Indian
- Arabic
- Indonesian
- Chinese
- Italian
- Eastern Europe
- Malaysian
- Greek
- New Zealander/Maori
- Hong-Kong
- Phillippine
- Horn of Africa
- Vietnamese

Other (please specify)

12. How would you describe your current living situation?

- Living alone.
- Living with friends/ housemates.
- Living with a partner.
- Living with a partner and my children.
- Living with my children.

13. Please choose the marital status that best applied to you.

- Never married
- Widowed
- Divorced
- Separated but not Divorced
- Married

How many children do you have?

____

14. What is the highest level of formal education you have completed?

- Did not go to school
- Primary school only
- Secondary school
- Tertiary Diploma
- Trade Certificate
- University Degree
- Postgraduate Degree

15. Please choose the employment status that best applies to you

- Working full-time
- Working part-time, casually, seasonally, or temping
- Unemployed
- Retired
- Household duties
- Receiving a pension/benefit
- Student Volunteer

Other (please specify)

____

16. Please choose the yearly income status that best applies to you.

- $0-9,999
- $10,000-19,999
- $20,000-29,999
- $30,000-39,999
- $40,000-49,999
- $50,000-59,999
- $60,000-69,999
- $70,000-79,999
- $80,000-89,999
- $90,000 or above

17. Do you smoke?

- Yes
- No

My physical activity behaviour

This survey examines different contexts in which you may be active.

SECTION 1: HOUSEHOLD AND FAMILY CARE ACTIVITIES.

First, we want to know about your activities at home, not including activities you may do at your home or other people's home for pay.

18. During the past year (12 months back from today), how much time did you spend...

	0- 1 hour a week	1 hour- 20 hours a week	20 + hours a week
Caring for a child or children between 2 and 5 years of age.			
Caring for a child or children under 2 years of age.			
Caring for a disabled child or elderly person (only count time actually spent in feeding, dressing, moving, etc.).			

19. During the past year (12 months back from today), how much time did you spend each day...

	0- 30mins	30 mins- 1hr	1- 1.5 hrs	1.5- 2hrs	More than 2 hrs
Preparing meals or cleaning up from meals on weekdays?					
Preparing meals or cleaning up from meals on weekends?					
Doing major cleaning, such as shampooing carpets, waxing floors, or washing walls or windows?					
Doing routine cleaning such as dusting, laundry, vacuuming, or changing linens?					
Going grocery shopping and pushing a shopping cart.					
Doing gardening or yard work, such as mowing lawn or raking leaves?					
Doing heavy outdoor work, such as chopping wood, tilling soil, shoveling snow, or baling hay?					
Doing major home decorations or repairs, such as plumbing, tiling, painting or building?					

20. Which best describes your current occupation?

- Employee of a private company, business or individual, for wages, salary or commissions.
- Government employee/ working for the public sector
- Self-employed in own business, professional practice or farm.
- Working without pay in a family business or farm
- Working without pay in the home (e.g. housework, childcare)
- I don't work (go to question 48).

What is your occupation?
(If more than one job, describe your occupation for the job with the most hours worked per week).

____

What kind of business or industry do you work in?
(For example, hospital, newspaper publishing, mail order house, university, call centre, etc.)

____

What specific tasks do you do most frequently in your occupation? (For example, typing, writing, managing people, etc.)

____

21. Please indicate that in comparison with other women your age, do you think your work is physically.....

- Much lighter
- Lighter
- The same as
- Heavier
- Much heavier

22. After work, are you physically tired?

- Never
- Seldom
- Sometimes
- Often
- Always

23. When you are working at your current occupation, how often do you do each of the following:

	Never	Seldom	Sometimes	Often	Always
Sit					
Stand					
Walk					
Lift heavy loads					
Sweat from exertion					

Active Living Habits

SECTION 3: ACTIVE LIVING HABITS

This section asks about the general level of physical activity involved in your daily routine during the past year.

24. How many minutes a day do you usually walk and/or bicycle to and from work, school or errands?

- Less than 5 minutes a day
- More than 5 minutes but less than 15 minutes a day
- More than 15 minutes but less than 30 minutes a day
- More than 30 minutes but less than 45 minutes a day
- More than 45 minutes a day

25. In the last year, how often did you watch television?

- Less than 1 hour a week
- More than 1 hour a week but less than 1 hour a day
- More than 1 hour a day but less than 2 hours a day
- More than 2 hours a day but less than 4 hours a day
- More than 4 hours a day

26. In the last year, how often did you walk (for at least 15 minutes at a time)?

- Never or less than once a month
- Once a month
- 2-3 times a month
- Once a week
- More than once a week

27. In the last year, how often did you cycle (for at least 15 minutes at a time?)

- Never or less than once a month
- Once a month
- 2-3 times a month
- Once a week
- More than once a week

Participation In Sports and Exercise

SECTION 4: PARTICIPATION IN SPORTS AND EXERCISE

Finally, we want to ask you about your participation in sports and exercise during the past year.

28. In comparison with other women your age, do you think you do... (Select most appropriate response)

- Much less physical activity
- Less physical activity
- Same amount of physical activity
- More physical activity
- Much more physical activity

29. In the past year, how often did you play sport or exercise?

- Never or less than once a month
- Once a month
- 2-3 times a month
- Once a week
- More than once a week

30. In the past year, how often did you sweat from exertion during sports or exercise?

- Never or less than once a month
- Once a month
- 2-3 times a month
- Once a week
- More than once a week

31. In comparison to your exercise routine before you reached menopause, do you sweat more or less when exercising now?

- Much less
- Less
- Same as
- More
- Much more

32. For each of the following sport or exercise activities, could you please indicate whether, during the past year, you have taken part in any of these activities or in any other similar activities not included in the list?

	Never	Not for a long time (not in the last year)	Not very often (occasionally in the year)	Sometimes (once or twice a month)	Regularly (once a week or more)
Running/Jogging					
Walking (more than one hour continuous)					
Cycling (more than 30 minutes continuous)					
Team sports (e.g. netball, basketball)					
Golf					
Horse Riding					
Racquet sports (e.g. squash, tennis, badminton)					
Dancing (e.g. line dancing, ballroom)					
Swimming					
Keep fit classes (e.g. aerobics, circuits)					
Bowls (indoor and outdoor)					
Gym (weight training or exercise machines)					
Boxing & Martial Arts (e.g. judo, karate)					
Aqua Aerobics					
Yoga, Tai-Chi, Pilates					

Any other activities you do which are not included in the above list (e.g. Did you do any other exercise or play any other sport in this past year).

Which sport or exercise did you do most frequently? (Specify ONLY ONE; If none please write “none”)

____

33. On a scale of 1-10, how much effort do you feel you put into this activity?

- 1 (No effort at all)
- 2
- 3
- 4
- 5
- 6
- 7
- 8
- 9
- 10 (A large amount of effort)

34. How many months in this past year did you do your most frequent activity?

- Less than 1 month
- More than 1 month but less than 3 months
- 4 to 6 months
- 7 to 9 months
- More than 9 months

35. How many hours a week did you usually do this activity?

- Less than 1 hour
- More than 1 hour but less than 2 hours
- More than 2 but less than 3 hours
- More than 3 but less than 4 hours
- More than 4 hours

What was the SECOND MOST frequent sport or exercise you did? (If none, write NONE):

____

36. How many months in this past year did you do your SECOND MOST frequent sport or exercise?

- Less than 1 month
- More than 1 month but less than 3 months
- 4 to 6 months
- 7 to 9 months
- More than 9 months

37. How many hours a week did you usually do this SECOND MOST frequent sport or exercise?

- Less than 1 hour
- More than 1 hour but less than 2 hours
- More than 2 but less than 3 hours
- More than 3 but less than 4 hours
- More than 4 hours

38. Have you intentionally avoided any activity due to your menopausal status? (eg. only tick yes, if your reasons for avoiding activity is solely due to your menopausal status).

- Yes
- No

Which activities have you actively avoided due to your menopausal status? (Please list all activities that you have actively avoided, eg. washing windows, playing netball).

What were your specific reasons for avoiding these activities? (eg. being hot; sweating too much; tiredness. Please list all reasons you can remember for avoiding activities).

How much my menopausal symptoms bother me?

39. Please indicate the extent to which you are bothered at the moment by any of these symptoms by selecting the appropriate number.

	Not at all	A little	Quite a bit	Extremely	N/A
Heart beating quickly or strongly.					
Feeling tense or nervous.					
Difficulty in sleeping.					
Excitable.					
Attacks of panic.					
Difficulty in concentrating.					
Feeling tired or lacking in energy.					
Loss of interest in most things.					
Feeling unhappy or depressed.					
Crying spells.					
Irritability.					
Feeling dizzy or faint.					
Pressure or tightness in head or body.					
Parts of body feel numb and tingling.					
Headaches.					
Muscle and joint pains.					
Loss of feeling in hands or feet.					
Breathing difficulties.					
Hot flushes.					
Sweating at night.					
Loss of interest in sex.					

What I am like?

40. In the questionnaire below are a number of statements that people often use to describe themselves. Please read each statement and then select the appropriate number next to that statement to indicate your answer. There are no right or wrong answers: Your own impression is the only thing that matters.

	False	Mostly False	Neutral	Mostly True	True
I make contact easily when I meet people.					
I often make a fuss about unimportant things.					
I often talk to strangers.					
I often feel unhappy.					
I am often irritated.					
I often feel inhibited in social interactions.					
I take a gloomy view of things.					
I find it hard to start a conversation.					
I am often in a bad mood.					
I am a closed kind of person.					
I would rather keep other people at a distance.					
I often find myself worrying about something.					
I am often down in the dumps.					
When socializing, I don’t find the right things to talk about.					
